# Organizational Leaders’ Views on Digital Health Competencies in Medical Education: Qualitative Semistructured Interview Study

**DOI:** 10.2196/64768

**Published:** 2025-03-07

**Authors:** Humairah Zainal, Xin Xiao Hui, Julian Thumboo, Fong Kok Yong

**Affiliations:** 1 Health Services Research Unit Singapore General Hospital Singapore Singapore; 2 Department of Rheumatology and Immunology Singapore General Hospital Singapore Singapore; 3 Duke-NUS Medical School National University of Singapore Singapore Singapore

**Keywords:** technology, medical education, curriculum, clinical competence, digital competence, Singapore, digital health, qualitative study, medical school, risk, comprehensive framework, doctor, thematic analysis, information technology, evidence-based, undergraduate, healthcare systems, mobile phone

## Abstract

**Background:**

Digital technologies (DTs) have profoundly impacted health care delivery globally and are increasingly used in clinical practice. Despite this, there is a scarcity of guidelines for implementing training in digital health competencies (DHC) in medical schools, especially for clinical practice. A lack of sustained integration of DHC risks creating knowledge gaps due to a limited understanding of how DT should be used in health care. Furthermore, few studies have explored reasons for this lag, both within and beyond the medical school curriculum. Current frameworks to address these barriers are often specific to individual countries or schools and focus primarily on curriculum design and delivery. A comprehensive framework is therefore required to ensure consistent implementation of DHC across various contexts and times.

**Objective:**

This study aims to use Singapore as a case study and examine the perspectives of doctors in organizational leadership positions to identify and analyze the barriers to DHC implementation in the undergraduate curriculum of Singapore’s medical schools. It also seeks to apply the Normalization Process Theory (NPT) to address these barriers and bridge the gap between health care systems and digital health education (DHE) training.

**Methods:**

Individual semistructured interviews were conducted with doctors in executive and organizational leadership roles. Participants were recruited through purposive sampling, and the data were interpreted using qualitative thematic analysis.

**Results:**

A total of 33 doctors participated, 26 of whom are currently in organizational leadership roles and 7 of whom have previously held such positions. A total of 6 barriers were identified: bureaucratic inertia, lack of opportunities to pursue nontraditional career pathways, limited protective mechanisms for experiential learning and experimentation, lack of clear policy guidelines for clinical practice, insufficient integration between medical school education and clinical experience, and poor IT integration within the health care industry.

**Conclusions:**

These barriers are also present in other high-income countries experiencing health care digitalization, highlighting the need for a theoretical framework that broadens the generalizability of existing recommendations. Applying the NPT underscores the importance of addressing these barriers to effectively integrate DHC into the curriculum. The active involvement of multiple stakeholders and the incorporation of continuous feedback mechanisms are essential. Our proposed framework provides concrete, evidence-based, and step-by-step recommendations for implementation practice, supporting the introduction of DHC in undergraduate medical education.

## Introduction

### Background

The integration of digital technologies (DTs) into clinical care is transforming health care worldwide [1], underscoring the need to prepare future health care professionals with digital health competencies (DHC) through digital health education (DHE). Despite widespread recognition of the importance of DHC, medical schools worldwide—including those in Singapore—have been lagging in their efforts to implement such training in a meaningful and systematic manner [2-8]. Countries like the United States, the United Kingdom, Canada, and Germany face similar challenges, such as fragmented integration efforts, limited faculty expertise, and curriculum overload, which hinder the consistent incorporation of DHE into undergraduate medical curricula [9-14]. Singapore, a high-income nation in Southeast Asia with advanced education systems and extensive digitalization, provides a compelling case study to explore these barriers. While Singapore’s unique sociopolitical and cultural context informs this study, the challenges it faces mirror those encountered by other high-income nations, highlighting the broader international relevance of this research.

Existing efforts to integrate DHE in medical schools, especially for clinical practice, are often disconnected, lacking systematic frameworks and sustained engagement with key stakeholders [2-8]. For example, in Europe and the United States, DHE initiatives are often siloed, leading to significant variability in the quality and scope of training [2,4,6]. Similarly, Australia has faced barriers such as a lack of standardized frameworks for digital health training and challenges in aligning medical education with rapidly evolving health care technologies [3].

These gaps result in inconsistencies in training, with DHC frequently treated as elective content rather than as a core component of medical education. This study applies Normalization Process Theory (NPT)—a framework designed to examine how new practices become embedded within institutions—to provide insights into systematically normalizing DHE and ensuring its sustainable integration [15].

This study addresses the following research questions: (1) What are the institutional and structural barriers to integrating DHE into undergraduate medical curricula? (2) How can the medical school experience be aligned with technological advances? (3) How can NPT be systematically applied to facilitate the effective and sustainable incorporation of DHE? By focusing on Singapore, this study not only provides a deeper understanding of these challenges but also offers insights that can inform global efforts to strengthen DHE integration in medical education.

### Exploring the Perspectives of Doctors in Organizational Leadership Roles

Despite their influence on governance and standards, the perspectives of doctors in organizational leadership roles are often overlooked. Existing research that evaluates the opinions of this group of stakeholders primarily addresses challenges in implementing DT in health care, characteristics of effective health systems, and key attributes for health care leaders [16-18].

This study recognizes that doctors in organizational leadership roles possess a strategic understanding of both clinical practice and medical education. Their insights are crucial as they can influence curriculum design, resource allocation, and policy formulation. By leveraging their dual perspectives, the study identifies unique barriers that may not be visible to frontline educators or students.

Furthermore, leaders in health care organizations have the authority to implement change and drive initiatives. Understanding their perspectives ensures that any proposed solutions are both feasible and likely to gain support at the highest levels of the institution. Their endorsement can facilitate smoother implementation and wider acceptance of DHE initiatives.

In addition, these leaders are often involved in broader system-wide decision-making. They are also likely familiar with challenges related to integrating new technologies and practices into established systems. Hence, their experience can provide valuable insights into overcoming any institutional inertia, aligning new initiatives with existing policies, and addressing systemic barriers.

### Medical Education in Singapore

Medical education in Singapore is provided by 3 schools, which are Yong Loo Lin School of Medicine (YLL) at the National University of Singapore (NUS), Lee Kong Chian School of Medicine (LKCMedicine) at the Nanyang Technological University (NTU), and Duke-NUS Medical School (Duke-NUS). YLL, established in 1905, and LKCMedicine, founded in 2013, both provide a 5-year undergraduate program. In this program, students spend 2 years studying the basics of medical sciences before undergoing clinical clerkships from the third to the fifth year. YLL was formed to address the critical health care needs of the local population during the colonial period, whereas LKCMedicine was established to meet the increasing health care demands due to an aging population [19]. To boost Singapore’s capabilities in translational medicine, Duke-NUS was founded in 2005 through a partnership between NUS and Duke University in the United States. Duke-NUS is a graduate medical school that offers a 4-year MD program, where the first year focuses on basic sciences and the second year on clinical postings. In their third year, students focus on developing research skills, and in their final year, they engage in clinical clerkships [20]. All medical schools in Singapore receive public funding, and students’ tuition fees are subsidized by the government.

Despite their cutting-edge facilities and innovation-driven educational technology, disparities persist between medical school training and clinical application [8,21]. Efforts to integrate DHE courses, such as virtual reality and point-of-care ultrasound, also vary in content and duration across these institutions [8]. Hence, standardizing the curriculum and ensuring consistent training across all medical schools is crucial to bridging the gap between theoretical knowledge and practical skills. This approach would enhance the overall competency of future health care professionals and improve the quality of patient care. In addition, ongoing assessment and adaptation of these programs to incorporate emerging technologies and methodologies are essential for keeping pace with the rapidly evolving medical field.

## Methods

### Data Collection

A qualitative study was conducted using individual semistructured interviews with doctors who are currently or have previously held organizational leadership positions. Participants were identified by our principal investigator (PI), FKY, based on their leadership roles within public health care organizations, ensuring they possessed the requisite knowledge and experience aligned with the research objectives. Selection criteria focused on senior leaders with expertise in research, clinical education, and development.

This cohort represented a niche group of chief physicians leading public tertiary hospitals that serve as teaching hospitals for undergraduate and postgraduate medical training. Specifically, participants included group chief executive officers from all 3 public health care clusters in Singapore, chairmen of medical boards at public health care institutions, senior administrators from the Ministry of Health, and Directors of Training and Education. To ensure a consistent depth of expertise, participants were required to have a minimum of 5 years of leadership experience within public health care organizations. Those with less than 5 years of such experience were excluded from the study.

Purposive sampling was used to ensure a diverse representation of organizational leaders based on factors such as organizational type, that is, public health care clusters and institutions and functional domains, ie, clinical services and administration. This approach enabled the collection of rich, varied perspectives, enhancing the study’s credibility.

Data collection took place from January to April 2021. Participants were invited by the PI by email, which provided a detailed outline of the study’s purpose, procedures, potential risks, and benefits. The email also included a consent statement for participants to review and acknowledge before proceeding. In reviewing issues of reflexivity, the threat of potential researcher biases due to the established professional relationship between the PI and the research participants was overcome by having the research fellow, who had no previous relationship with any of the participants, as the interviewer. During the Zoom (Zoom Video Communications) interview, participants were given the opportunity to ask questions, and their verbal consent was recorded at the beginning of each session to ensure informed and voluntary participation. They were reminded of their right to withdraw from the study at any point. It was clarified that data collected prior to withdrawal would still be retained and analyzed to enable a comprehensive evaluation of findings.

To protect participants’ anonymity, we assigned code identifiers beginning with “OL” (organizational leader) to each of them. Any identifying information and audio recordings were stored separately from the main dataset in a secure, password-protected file, accessible only to authorized research team members. In reporting results, care was taken to remove or generalize any details that could potentially identify individuals. The data collected and analyzed was used exclusively to inform curriculum development, with no intention to disclose identifiable information.

The interview guide was developed based on the NPT constructs of coherence, cognitive participation, collective action, and reflexive monitoring (Textbox 1). We then adapted the interview guide iteratively to allow participants to share their views on matters that were not initially included in the guide. Generally, the questions sought participants’ views on the clinical skills that are still relevant in the digital age (coherence), additional skills that medical students and doctors need for clinical practice amid increasing health care digitalization (coherence), and the clinical skills that are currently being covered in local medical schools (coherence). We also asked participants for their opinions on the clinical skills that should be emphasized more in the medical school curricula (coherence), the challenges of integrating DHE into the compulsory curricula, and suggestions for curriculum improvement to better prepare students for future clinical practice (cognitive participation). To explore how “Collective Action” could be operationalized, we asked how medical schools could improve their collaboration with other stakeholders, particularly professional bodies, health care institutions, and the health care system, to better prepare medical students for clinical practice in the digital era (collective action). We also raised the question of how participants would evaluate the impacts, benefits, and areas for improvement of DHE initiatives within the medical school curriculum (reflective monitoring).

**Interview questions.**

- In general, what are the clinical skills that a medical doctor should have?
- Which of these skills are still relevant in the digital age?
- Are there any skills that have been replaced by digital technology, be it partially or completely?
- Against the backdrop of increasing digitalization of health care, what new skills, clinical or otherwise, should a doctor have in order to practice medicine?
- What clinical skills are currently being covered in the local medical schools?
- Which of these skills should be emphasized more in the medical school curriculum?
- In your opinion, how well do the current medical school curricula prepare medical students for the digital aspects of health care? What do you think are some of the challenges in implementing digital health education in medical schools?
- What other improvements can be made to our local medical school curriculum to better prepare the students for clinical practice in light of rapid advances in technology (for example, the advent of artificial intelligence, big data, imaging, smartphone applications, and digital equipment such as handheld ultrasound)?
- How can local medical schools improve their collaborations with professional bodies and health care institutions to prepare medical students for clinical practice in this era of new technology?
- What can the health care system do to support medical students and young doctors in this era of new technology?
- Do you have any other comments on the digital transformations of medicine or health care before we end this interview?

Challenges of contextual differences and stakeholder variation are crucial factors that need to be carefully considered when applying NPT in diverse settings. The study was conducted in Singapore, where the adoption of DT within health care settings has been gradual [7]. This presents challenges for students who may not have adequate exposure to digital systems during their clinical placements. In response to this, the application of NPT should be focused on building digital literacy and ensuring that any intervention is compatible with ongoing efforts to integrate digital solutions into clinical practice. Furthermore, there is also a limited innovation culture in Singapore’s health care system [7]. To overcome this, interventions that adopt NPT should incorporate elements designed to stimulate collaboration and mentorship programs with industry professionals. This would help bridge the gap between academic training and the innovation needs of the health care sector.

The study included 33 participants, with the sample size determined based on theoretical and practical considerations. Data collection continued until saturation was reached, ensuring that no new themes or insights emerged from the interviews. This indicates that the sample size was sufficient to capture the relevant perspectives for the study. While practical constraints, such as time and resources, influenced the final number of participants, the primary focus was on ensuring data richness and diversity. This approach allowed for a comprehensive exploration of the research questions.

A total of 30 interviews were conducted and recorded over Zoom due to the physical restrictions brought about by the COVID-19 pandemic, while 3 in-person interviews were held with participants who were located in areas with fewer restrictions at the time or who specifically preferred in-person interaction. All in-person interviews were carried out in accordance with local health guidelines to ensure participant safety. Each interview lasted approximately 40 minutes and was audio-recorded. The transcriptions were derived from the audio recordings of the interviews, which were processed using Otter.ai software (Otter.ai, Inc) before being reviewed for accuracy by the PI and research fellow.

### Data Analysis

Thematic analysis using Braun and Clarke’s [22] 6-step framework was used to explore barriers that emerged from the data, while a deductive approach based on the constructs of NPT was used to map suggestions for curricula improvement to relevant NPT constructs. To overcome potential interpretive bias and selective perception, coding was conducted by 2 researchers independently. After the initial coding, discrepancies were discussed, and a consensus was reached to refine the codebook and ensure consistency in the application of codes. To enhance credibility and trustworthiness, data were triangulated by comparing the findings across participants from various public health care clusters to identify any consistencies and divergences in opinions. This helped to ensure that the themes captured diverse perspectives and were not unduly influenced by any single group.

In addition, we contextualized the findings by examining studies from other high-income countries undergoing similar digital transformations in health care. Furthermore, we analyzed recently published data reflecting the perspectives of other stakeholders in the health care industry, such as clinical educators and leaders of medical schools, regarding the digital competencies required for future clinical practice [8,21]. In the reporting of findings, we followed the Standards for Reporting Qualitative Research of O’Brien et al [23].

### Ethical Considerations

This study was classified as a quality improvement (QI) project focusing on medical education curricula by the Research Integrity, Compliance, and Ethics (RICE) committee of SingHealth. In line with institutional guidelines, QI projects aimed at enhancing existing practices, processes, or programs, such as curriculum development in medical education, do not meet the criteria for human subjects research. As such, the study was granted an ethical waiver by the SingHealth Centralized Institutional Review Board (2020/2880). This decision was based on the determination that the activities involved posed no more than minimal risk to participants. Despite this waiver, the research adhered strictly to the ethical principles outlined in the World Medical Association’s Declaration of Helsinki and institutional guidelines.

## Results

A total of 33 participants took part in the study. They included 19 chief medical officers from local public health care institutions, 3 chief executive officers from public health care clusters, 4 senior administrators, and 7 former organizational leaders. Each had at least 5 years of organizational leadership experience and represented various specialties (Table 1).

Participants shared that local medical schools have not yet revamped the curricula to incorporate relevant competencies for the digital age. They identified 6 reasons for the lag in DHC training, some of which extended beyond the medical schools. The analysis of codes, along with the generation of subthemes and themes, is summarized in Table 2. Illustrative quotes from the interviews are provided below.

**Table 1:** Demographics of participants (N=33).

Characteristics			Participants
**Age (years)**			
	Mean	62	
	Median	60	
	Minimum age	44	
	Maximum age	82	
**Gender, n (%)**			
	Male	31 (94)	
	Female	2 (6)	
**Years in organizational leadership**			
	Mean	18.7	
	Median	18	
**Discipline, n (%)**			
	Gastroenterology and hepatology	5 (15.2)	
	Pediatrics (including pediatrics genetics, pediatric emergency medicine, and pediatric gastroenterology)	4 (12)	
	General surgery	3 (9.1)	
	Psychiatry	3 (9.1)	
	Renal medicine	3 (9.1)	
	Anesthesiology	2 (6.1)	
	Geriatric medicine	2 (6.1)	
	Respiratory medicine	2 (6.1)	
	Cardiology	2 (6.1)	
	Orthopedic surgery	2 (6.1)	
	General medicine	1 (3)	
	Medical oncology	1 (3)	
	Ophthalmology	1 (3)	
	Surgery and urology	1 (3)	
	Hand and reconstructive microsurgery	1 (3)	

**Table 2:** Codes, subthemes, and themes identified from the coding process.

Codes	Subthemes	Themes
Lack of time. Hard to change. Resistance. Not open to new technologies. Not willing to try new technologies. Academics have to be open.	Packed curriculum. Preference for status quo. Traditional mindset of senior clinicians and faculty.	Bureaucratic inertia.
Lack of alternative career pathways. Lack of role models. Mindset changes needed.	Expectations for graduates to become doctors with patient-fronting roles.	Limited opportunities to pursue traditional career pathways.
Safe. Safe sandbox. Safety nets. Patient safety. Safe and creative space. Nurture and protect. Talk about the pitfalls and dangers of using technology.	Lack of safety mechanisms to use DT^a^ for educational purposes. Limited opportunities to experiment with new technologies due to lack of creative space.	Lack of protective mechanisms for experiential learning and experimentation.
Clear guidelines. Clear policies. Clear intent. Clear boundaries. Help students navigate data, fake news, and misinformation. Data abuse. Medical ethics. Respect privacy. Ethical competency. Schools presume these (ethical competencies) are common sense.	Gaps in outlining guidelines and boundaries for technology use. Gaps in teaching students the pitfalls of using technologies for clinical practice. Gaps in equipping students with skills in handling data, medical information, and patients’ privacy.	Lack of clear policies and guidelines for clinical practice.
Interface. Incorporate teaching facilities within health care institutions. Correlate. String information.	Limited integration of educational and research facilities for medical students within clinical settings. Lack of feedback on students’ performance outcomes. Lack of compatible data encountered in medical school and residency.	Lack of integration between medical school education and experience in the health care system.
Gap between IT and health care. Nonintegration. Disorganized. Slave to the system. Need to redesign the system. Put up robust systems. Involve IT experts. Facilitating platforms. Support end users. Internet separation.	Health care industry should drive the IT industry.	Lack of IT integration within the health care industry.

^a^DT: digital technology.

### Bureaucratic Inertia

Participants suggested that bureaucratic inertia within both the health care system and medical schools contributed to sporadic and limited training in DT. They attributed this inertia to faculty members' lack of awareness regarding the evolution of clinical practice, their limited expertise in DT, and their resistance to incorporating new competencies, which would require sacrificing some traditional areas of expertise. As shared by OL8 and OL26:

There are senior clinicians who may not be so open to using DT. They are not willing to use different methodologies to solve the same problem.

OL8, Internal medicine, and Respiratory and Critical Care Medicine

I tried to teach ultrasound in a medical school but with limited success… Unfortunately, it was met with great resistance from people who are traditional.

OL26, Cardiology

Furthermore, participants perceived that policy makers and senior clinicians were hesitant to invest in DT due to concerns over higher health care costs, further hindering efforts to optimize DT in clinical settings. This perspective is illustrated by the following comment:

Some new technologies are almost invariably more expensive and will increase the cost of care.

OL4, General Surgery

The above excerpts highlighted systemic barriers to the integration of DT in clinical practice and medical education, emphasizing how institutional inertia and hesitation to invest in new technologies are contributing to the stagnation in clinical training and practice. The reluctance of policy makers and leaders to embrace change and allocate resources for DT exacerbates these challenges, ultimately hindering the evolution of medical education.

### Lack of Opportunities to Pursue Nontraditional Career Pathways

Participants also identified limited opportunities to pursue alternative career paths and nonclinical roles, as well as the absence of role models in new technology fields, as significant barriers to implementation. As opined by OL26:

I’ve seen promising students and residents fall through the cracks and give up along the way because we don’t have enough career pathways and role models for those in the medical innovation track.

OL26, Cardiology

In addition, OL8 highlighted the stigma within the medical community, where students who left medical school to explore nontraditional pathways were often perceived as failures.

We lack the definition of what kind of medical graduates we want to train. Other than basic clinical knowledge, I don’t think we have defined anything further than that, like a clinician with knowledge of innovation. If a student decides to be an entrepreneur, for example, create a new start-up and drop out of medical school, we should still take that as a success and not a failure.

OL8, Internal Medicine and Respiratory and Critical Care Medicine

Without embracing alternative career paths and addressing the stigma associated with leaving traditional medical roles, the health care system risks alienating promising talent and limiting progress in medical innovation. Establishing clear pathways and celebrating diverse career outcomes is essential to cultivating a dynamic and adaptable health care profession.

### Lack of Protective Mechanisms for Experiential Learning and Experimentation

In addition, participants noted limited protective mechanisms for experiential learning and experimentation in the health care system. The lack of a “safe and creative space” hindered trainees from engaging in innovative and secure experimentation with DT. Some participants proposed establishing sandboxes where trainees could test ideas with safeguards in place. This would enable them to contribute to clinical practice improvements while receiving proper guidance when mistakes occur. As articulated by OL8 and OL9:

The senior clinicians may not be so open to new things. As health care leaders ourselves, we need to embrace the idea of creating a safe sandbox where students [are] allowed to use their imagination to innovate, with all the safety nets in check for patient safety.

OL8, Internal Medicine, and Respiratory and Critical Care Medicine

What’s lacking is a safe space for students and residents. A safe space is a space that offers professional, psychological, and personal safety for them. Measures need to be taken to train, nurture, and protect them rather than condemn them when they do something wrong. The health care system should give them that safe and creative space that ensures they are not bullied, harassed, and ridiculed.

OL9, Anesthesiology

Without the establishment of structured and supportive environments for experiential learning, the health care system risks stifling innovation and deterring the next generation of clinicians from engaging with DT. Proactively establishing protected and guided learning environments is essential for fostering a culture of experimentation and ensuring meaningful contributions to clinical advancements.

### Lack of Clear Policies to Guide DT Integration in Clinical Practice

Another significant barrier articulated by participants was the lack of clear policies to guide the effective integration of DT in clinical practice. They emphasized the need for well-defined guidelines at both institutional and ministerial levels to support the ethical and professional use of DT. As noted by OL11:

The policies that govern digital technologies like telemedicine must be reasonable. Currently, the intent is unclear. At the institutional and ministerial level, there must be clear guidelines and policies that outline the learning and growth in the use of these technologies.

OL11, Geriatric Medicine

The lack of comprehensive policies limits awareness of the risks, pitfalls, and ethical considerations associated with DT, deterring its use, particularly among students. OL25 elaborated on the importance of training students in ethics and professionalism to prevent potential misuse of data.

In the world of AI and digital medicine, the role of ethics and professionalism are going to be even more important because it opens up easy channels to data abuse, and doctors will have so much data in their hands. So, you need to teach the students medical ethics and values related to patient information and treatment prescription. It’s going to be so critical you need to enforce that.

OL25, Medical Oncology

The absence of clear, comprehensive policies to govern the use of DT in health care creates ethical and professional ambiguities, deterring adoption and proper training. Establishing well-defined guidelines is critical to mitigating risks, ensuring ethical use, and preparing future clinicians to navigate the complexities of digital medicine responsibly.

### Lack of Integration Between Medical School Education and Clinical Experience

Participants shared that the perceived lack of integration between medical school education and students’ clinical experience in the health care system is another barrier to DHE. They attributed this gap to the lack of systems interoperability, which prevents students from accessing and using health care data used in clinical settings and receiving feedback from these systems. As one participant explained, a more integrated system would allow student performance data to correlate with hospital data, enabling continuous feedback and supporting learners’ improvement:

The biggest gap is that we don’t know how students are performing. The data that students are trained for should be similar to the place of practice. If the system is built such that medical school data correlates with say, hospital data, I can string all the information about your learning journey and see how that impacts your performance outcome. From that perspective, we can support the learners better because we give them an environment where they are constantly receiving feedback from the system and seeking new ways to improve themselves. I think that will probably be the most meaningful thing for our learners.

OL15, Psychiatry

More broadly, participants noted that the lack of integration between educational and research facilities within health care institutions limits students’ clinical immersion. According to OL16, closer collaboration between medical schools and health care institutions is essential for strengthening this connection and enhancing experience, not just physically but through more active interaction between the institutions and health care professionals.

I think medical schools should be in the health care institutions. They should interface very closely. One way is to incorporate teaching and research facilities within health care institutions so that the immersion is useful. Currently, our medical schools are within the proximity of the hospital campus. It makes sense, but that’s just the physical infrastructure. The people need to be interfaced quite a fair bit.

OL16, Psychiatry

The fragmented nature of medical education and clinical training suggests that a more integrated approach, leveraging data-driven feedback mechanisms and collaborative partnerships between academic and health care institutions, is necessary to foster a culture of continuous learning and improvement in health care.

### Lack of IT Integration Within the Health Care Industry

Participants also suggested that an integrated IT infrastructure in health care institutions would increase DHE effectiveness and enhance clinical care. However, they highlighted the current lack of interoperability between systems, which hinders the optimization of technical needs. A recurring concern was the IT sector’s lack of ability to understand and address the specific needs of health care, with participants noting disorganization and a disconnect between IT and health care practices. As one participant expressed:

The gap between the IT and health care industry has not been bridged yet. We have a lot of IT in the health care industry, but a lot of it is record-keeping. It does not integrate [and] information is coming from every direction that is totally disorganized. How, then, can we teach our medical students to be responsible for the patient as a whole? Somebody who has the ability to do IT programming has to follow the doctors on their rounds. I’ve ever asked my IT colleagues, “Look, is this an IT industry or a health care industry? When they said it’s a health care industry, I said, okay, then you have to listen to me and make things work for me, not enslave me to your products.”

OL9, Anesthesiology

Furthermore, participants emphasized the challenge of internet separation and the need for platforms that allow seamless cross-sharing of information, which they identified as crucial for effective learning environments. As shared by OL12:

One of the biggest challenges is Internet separation…The availability and cross-sharing of information are all important facilitating platforms that we have to provide for medical students.

OL12, Pediatrics

The lack of integrated IT infrastructure and disjointed systems within health care settings creates significant barriers to enhancing DHE and clinical care, often leaving medical practitioners frustrated with ineffective solutions. To bridge the gap between IT and health care, a more tailored approach is needed, where technological systems are designed to directly support clinical workflows, ensuring both efficiency and improved educational outcomes.

## Discussion

### Principal Findings

By interviewing doctors in organizational leadership, we gained insider perspectives on gaps in both the medical curricula and the health care system. A total of 6 barriers were identified: bureaucratic inertia, lack of opportunities to pursue nontraditional career paths, limited protective mechanisms for experiential learning, unclear policy guidelines, limited integration between education and clinical experience, and IT integration issues. The findings contributed to the existing literature by showing that DHE barriers were not limited to medical school curricula but involved broader systemic issues. Comprehensive strategies were needed to address these challenges.

By using qualitative interviews, our study uncovered nuances in leadership decision-making that are often missed in quantitative surveys, providing a richer understanding of the factors influencing leadership perspectives. While most studies suggest that organizational leaders prioritize efficiency and sustainability [16-18], our findings reveal that leaders in this context place a higher emphasis on experimentation and innovation, a factor not traditionally associated with corporate leadership. Furthermore, our research also highlights the growing influence of digital transformation on leadership styles, an area that received limited attention in previous studies focused on traditional management structures. It underscores the importance of adaptive leadership in an era of constant change, suggesting the need for leadership training programs that focus on flexibility.

Many of the barriers identified in this study align with findings from other high-income countries. These include the lack of the necessary information and communication technology (ICT) skills and limited awareness of the potential benefits of DT among some clinicians. For example, in Germany, an empirical study by Ernstmann et al [24] revealed that some primary care doctors perceived eHealth cards as less useful due to their limited ICT expertise and lack of involvement in technological development. These eHealth cards, which store medical data, treatment plans, medications, and electronic patient files, rely on a telematics infrastructure for communication [24]. The study recognized that without robust IT support, comprehensive training for medical professionals, and a standardized national implementation procedure, the acceptance, adoption, and sustained use of eHealth technology by doctors are likely to be hindered [25].

In addition, other studies have shown an increasing proportion of medical school graduates pursuing careers outside full-time clinical practice in some countries [26]. However, findings from countries such as the United States and South Korea indicate that medical school curricula often fail to adequately address the need for programs providing information on nontraditional careers or nonclinical career pathways [27,28]. Despite expressed interest in these career options, medical students often lack awareness of available training opportunities. To attract students to such careers, early outreach programs, combined with appropriate indemnity and support for innovative projects, are essential. These initiatives could be implemented through elective classes, incentives from professional societies, or partnerships with experts [27].

Furthermore, research from countries such as Canada and Taiwan highlights how technological tools can be leveraged to foster experiential learning among medical students. At the University of Ottawa, social accountability experiential logs were developed for third-year medical students to address the social determinants of health, which are often overlooked in clinical learning objectives [29]. These logs guided students in reflecting on clinical encounters and targeting psychosocial skill development, improving clinical confidence, and demonstrating adaptability for other medical schools (Fung et al [29]). Similarly, a Taiwanese study by Liao et al [30] showcased how the mPath (KU Leuven) e-learning tool supported communication skills training by providing a flexible, technology-enhanced learning environment [30]. Features such as remote accessibility, session recordings, peer feedback mechanisms, and visualized analytical reports enabled learners to engage in self-reflection, adapt communication strategies, and enhance subverbal communication skills [30]. Together, these initiatives exemplify how experiential learning tools can address both biomedical and psychosocial challenges in medical education.

The lack of clear laws and policies to guide DT integration in clinical practice is also a barrier in other high-income countries. For instance, health care leaders in Sweden have acknowledged the need for updated policies [16]. They noted that existing laws and regulations have not kept pace with rapid technological advancements and the evolving organization of health care. These policies require revision to ensure clarity regarding liability and accountability, particularly in addressing how errors are managed when artificial intelligence (AI) systems play a role in clinical decision-making [16].

Furthermore, the limited integration between medical education and clinical experience has been highlighted in various studies and reviews. For instance, Pereira et al [31] describe the implementation of a single competency-based Epic onboarding process for medical students in certain US medical schools with rotations across multisystem training sites. This initiative has enabled learners to spend more time in clinical settings with optimized access to electronic health records (EHRs) [31]. While this approach reduces the training burden, curricula could be further enhanced by emphasizing the practical application of EHRs in clinical settings. This includes training students to maintain professionalism and establish rapport with patients while using EHR systems [31]. In addition, Chan and Zary [32] emphasize that providing immediate and formative feedback on students’ performance can support the effective use of AI in medical education. However, delivering high-quality feedback in clinical contexts remains a challenge, as it depends on the underlying knowledge base and model of the AI system, which still requires refinement [32].

Previous systematic reviews have consistently identified infrastructure and technical barriers as the most frequently cited barriers to technology integration in health care [33]. These challenges include limitations in health care capacity for technology adoption, inadequate interconnectedness, insufficient network resources, and incompatibility with existing daily workflows [33]. Addressing these barriers requires the active involvement of health care professionals in the development and implementation of health technology tools, which can also enhance their capacity to effectively manage such applications. Furthermore, the reviews emphasize the critical importance of user engagement and collaboration with system developers throughout all phases of design, development, deployment, and continued use [33]. This collaborative approach ensures that the applications are fit for purpose, as they are designed to align with and address health care providers’ needs and expectations.

Our findings highlighted structural and bureaucratic barriers beyond medical schools that hindered DHE implementation. Although they are common in high-income countries, no comprehensive framework has been proposed to address them to date. This study applies May and Finch’s [15] NPT to suggest ways to bridge these gaps. A summary of how the 4 constructs of NPT can be applied to each of these barriers is found in Table 3.

**Table 3:** Addressing each identified barrier with the Normalization Process Theory (NPT).

Barriers	NPT contributions
Bureaucratic inertia	Coherence: enhance understanding and sense-making among stakeholders about the importance and benefits of DHC^a^. This could be achieved by hiring prospective faculty with the skill sets that are relevant to the needs of up-and-coming developments in medicine. Cognitive participation: engage key stakeholders to foster buy-in and commitment. For example, leaders of medical schools can engage individuals with influence to encourage the integration of digitalization in the core curriculum. These include engaging clinical educators, teachers, and innovators trained in DT^b^ in knowledge exchange and talking with the faculty to facilitate the training of DHC and keep them abreast of the latest technological developments in clinical settings. Collective action: develop strategies to streamline decision-making processes and reduce red tape. Reflexive monitoring: continuously evaluate and adjust strategies to address bureaucratic resistance and demonstrate early successes to build momentum.
Lack of opportunities to pursue nontraditional career pathways	Coherence: clarify the relevance of DHC to future career opportunities and the evolving landscape of health care. Cognitive participation: involve influential faculty and practitioners, such as medical innovators, in promoting the value of alternative career pathways. Medical schools should also provide sufficient training and mentoring opportunities for students who wish to pursue alternative career pathways. Sufficient resources should be invested in implementing a curriculum that provides students with opportunities to diversify their skill sets, such as skills in clinical informatics relevant to clinical practice. It should include collaborative mentorship where students can explore new fields in DT by forming partnerships with experts from both the clinical and nonclinical fields. Collective action: integrate DHC into career development programs and highlight role models who have successfully incorporated digital skills. Reflexive monitoring: gather feedback from students and professionals to continually refine the approach and address concerns about career impact. Relevant recognition should also be given to medical graduates who embark on alternative pathways to encourage the growth of the fields and normalize these pathways for them.
Lack of protective mechanisms for experiential learning and experimentation	Coherence: emphasize the importance of experiential learning for mastering DHC. Cognitive participation: foster a culture of experimentation and learning by involving faculty in the design and delivery of experiential learning opportunities. Collective action: develop and implement policies and resources that support protected time and space for experiential learning and innovation. These include creating more sandboxes and expanding reasonable access to EHRs in clinical settings. Reflexive monitoring: continuously assess and improve experiential learning programs based on feedback and outcomes.
Lack of clear policy guidelines for clinical practice	Coherence: clearly articulate the need for and benefits of standardized DHC policies. Cognitive participation: engage policy makers, clinical leaders, and educators in developing and endorsing clear guidelines. To ensure that the threat of litigation does not hinder technological adoption, professional bodies should establish clear policies that regulate the effective implementation of DT in clinical settings. A technology assessment committee could also be set up to develop guidelines that enable young trainees to use DT effectively and ethically, both for their safety as well as for their patients. Collective action: implement training and support systems to ensure consistent application of policies across clinical settings. Professional bodies should also work with schools to equip students with knowledge of cybersecurity as well as the limitations and pitfalls of using DT in various circumstances. Reflexive monitoring: regularly review and update policies based on clinical practice feedback and emerging best practices. Dedicating time to reflect on what can be improved along the way would be a crucial step for schools.
Insufficient integration between medical school education and clinical experience	Coherence: highlight the importance of integrating DHC across the continuum of medical education. Cognitive participation: involve both academic and clinical faculty in designing integrated curricula that seamlessly blend theory and practice. Collective action: develop joint academic-clinical initiatives and placements that reinforce DHC training in real-world settings. Reflexive monitoring: evaluate the effectiveness of integrated programs and make adjustments to enhance alignment between education and practice.
Limited IT integration within the health care industry	Coherence: communicate the critical role of IT in supporting DHC and improving health care outcomes. Cognitive participation: collaborate with IT professionals and health care administrators to prioritize IT integration. To ensure that digital health care technologies can be used safely and effectively by clinicians, new technology or equipment introduced for clinical practice needs to be installed by IT personnel with knowledge of the health care system and with input from health care professionals so that the latter’s needs are met. Collective action: advocate for investments in IT infrastructure and training to support DHC initiatives. At the national level, a move towards interoperability of systems that allow users to share data would also facilitate students’ adaptation to new systems in different health care settings. Reflexive monitoring: continuously assess the state of IT integration and address gaps through ongoing improvement efforts.

^a^DHC: digital health competencies.

^b^DT: digital technology.

### Limitations of the Study

This study has several limitations that should be acknowledged. First, the focus on the perspectives of organizational leaders may not fully represent the experiences of frontline educators or students, limiting the generalizability of the findings. Furthermore, interviewing organizational leaders may introduce a bias toward presenting their organizations in a favorable light. They may be reluctant to express views that could be perceived as critical of their organizations. This concern may stem from the constraints they feel due to their roles or the public image of their organizations. As a result, their responses might reflect a more measured or politically cautious perspective. To address this, we incorporated triangulation by cross-referencing their responses with published articles on similar topics. This approach provided a more balanced perspective, though we recognize the inherent limitations in capturing the full organizational dynamics.

Second, the relatively small sample size, while sufficient to achieve thematic saturation, may constrain the breadth of insights. We also recognize that the unique sociopolitical, cultural, and economic context of Singapore may limit the generalizability of our findings to other settings. Singapore’s centralized governance and relatively small population create conditions that may differ from other countries. Consequently, while the insights from our study provide valuable lessons, they should be interpreted with caution when applying them to contexts with different governance structures or cultural dynamics.

The third limitation was the gender imbalance among the organizational leaders interviewed, with 94% (31/33) male and only 6% (2/33) female participants. While this reflects the current leadership demographics within public health care institutions, the barriers and challenges identified in our research are rooted in institutional and structural factors rather than individual-level or gender-specific experiences. As such, we do not expect that the gender distribution significantly influenced the findings. However, future research could benefit from a more gender-diverse sample to explore whether different leadership perspectives might offer additional insights or nuances.

Another limitation of our study is the use of Zoom for interviews, which was necessitated by the COVID-19 pandemic and which might have influenced the depth and dynamics of the discussions compared to in-person interviews. In face-to-face settings, nonverbal cues such as body language, eye contact, and physical proximity play a significant role in building rapport and fostering a more comfortable environment for in-depth conversations. These subtle cues can often provide valuable insights into a participant’s emotional state, engagement level, and willingness to share more personal or sensitive information. Nonetheless, the insights obtained through Zoom still offer valuable contributions to understanding the barriers to DHE integration.

In addition, we acknowledge that the NPT’s focus on individual experiences may not fully capture the diversity of perspectives of multiple stakeholders. To address this, we triangulated our data by comparing the findings across participants from various public health care clusters to identify any consistencies and divergences in opinions. This helped to ensure that the normalization process was not unduly influenced by any single group. By addressing these challenges, we believe our study provides a more nuanced understanding of NPT, particularly in contexts where contextual variations and diverse stakeholder groups are at play. These adaptations strengthen the applicability of NPT and offer valuable insights for its broader use in similar settings.

While we have made considerable efforts to adapt NPT to our specific context, we recognize that there may still be limitations in generalizing our findings across very different settings. Thus, future research should explore how NPT applies in more varied environments with larger sample sizes to further validate our findings. Furthermore, given that normalization is a gradual process, further studies should also conduct longitudinal follow-up assessments to monitor changes over time.

### Strengths of the Study

This qualitative study informs us about the institutional and structural barriers present in Singapore’s medical school curricula. The diverse sample of this study, spanning various health care institutions and specialties, yielded rich data. Participants possessed extensive organizational leadership experience and were attuned to the needs of contemporary clinical practice. Unlike previous research focusing mainly on institutional inertia and pedagogical strategies [5,34-37], this study uncovered structural barriers as well.

While findings may seem limited to Singapore’s context, applying relevant NPT constructs could render results applicable globally since many other high-income countries faced similar challenges in technological development and curriculum digitalization [3,12,38]. Furthermore, the identified barriers necessitated universal solutions extending beyond Singapore.

A potential line of future research would be to gather the views of medical innovators and entrepreneurs to explore other barriers to the effective adoption of DT in health care institutions. Another area would be to evaluate the ways in which DHC training among medical trainees and graduates influences the efficiency and cost-effectiveness of health care delivery. This research could provide valuable insights into how DHC in medical education affects not only the preparedness of new health care professionals but also the overall performance of health care organizations.

### Conclusions

Focusing on the perspectives of doctors in organizational leadership roles provides a comprehensive understanding of the barriers to incorporating DHE into Singapore’s medical curricula. Their strategic insight, policy influence, experience with system-wide challenges, understanding of the education-practice gap, resource management capabilities, and expertise in innovation and change management are invaluable for developing practical, effective, and sustainable strategies to address these barriers.

Unlike previous studies focusing solely on gaps within schools, our findings underscored the importance of collaborations with professional bodies and health care institutions to overcome various barriers. By applying NPT, this study provides a structured approach to understanding and overcoming the barriers. It offers a roadmap for other countries facing similar challenges in DHE. However, NPT should be seen as adaptable, requiring regular reevaluation to accommodate dynamic changes in the field.

## Abbreviations

AI: artificial intelligence
DHC: digital health competencies
DHE: digital health education
DT: digital technology
EHR: electronic health record
ICT: information and communication technology
LKCMedicine: Lee Kong Chian School of Medicine
NPT: Normalization Process Theory
NTU: Nanyang Technological University
NUS: National University of Singapore
OL: organizational leader
PI: principal investigator
QI: quality improvement
RICE: Research Integrity, Compliance, and Ethics
YLL: Yong Loo Lin School of Medicine
